# The impact of pH and temperature on the green gold nanoparticles preparation using Jeju Hallabong peel extract for biomedical applications†

**DOI:** 10.1039/d4ra00614c

**Published:** 2024-05-03

**Authors:** Ridhola Tri Ariski, Kyung Kwan Lee, Yongkwan Kim, Chang-Soo Lee

**Affiliations:** a Bionanotechnology Research Center, Korea Research Institute of Bioscience and Biotechnology (KRIBB) Daejeon 34141 Republic of Korea ariskiridh@gmail.com lkk@kribb.re.kr cslee@kribb.re.kr; b Department of Biotechnology, University of Science & Technology (UST) Daejeon 34113 Republic of Korea; c Wildlife Disease Response Team, National Institute of Wildlife Disease Control and Prevention (NIWDC) Gwangju 62407 Republic of Korea kyk5388@korea.kr

## Abstract

The utilization of gold nanoparticles (AuNPs) has garnered significant attention in recent times, particularly in the field of biomedical research. The utilization of AuNPs in chemical synthesis procedures raises apprehensions regarding their potential toxicity in living organisms, which is inconsistent with their purported eco-friendly and cost-effective aspects. In this investigation, AuNPs were synthesized *via* the green synthesis approach utilizing Jeju Hallabong peel extract (HPE), a typical fruit variety indigenous to South Korea. The visible-range absorption spectrum of gold nanoparticles from green synthesis (HAuNPs) that are red wine in color occurs at a wavelength of *λ* = 517 nm. The morphology and particle size distribution were analysed using transmission electron microscopy (TEM) and ImageJ software. The TEM images reveal that the HAuNPs exhibit a high degree of dispersion and uniformity in their spherical shape, with an average size of approximately 7 nm. Moreover, elevating the initial pH level of the mixed solution has an impact on the decrease in particle dimensions, as evidenced by the blue shift observed in the UV-visible spectroscopy absorbance peak. Elevating the reaction temperature may accelerate the synthesis duration. However, it does not exert a substantial impact on the particle dimensions. The outcomes of an avidin–biocytin colorimetric assay provide preliminary analyses of possible sensor tunability using HAuNPs. The cytotoxicity of HAuNPs was evaluated through *in vitro* studies using the MTT assay on RAW 264.7 cell lines. The results indicated that the HAuNPs exhibited lower cytotoxicity compared to both chemically reduced gold nanoparticles (CAuNPs).

## Abstract 2

Promising AuNPs were synthesized through reduction of gold chloride salt (Au^3+^) to gold nanoparticles (Au^0^) is carried out by phenolic compound as a reduction agent and flavonoid molecules as stabilizing agents by utilizing Jeju Hallabong peel extract.

## Graphical abstract

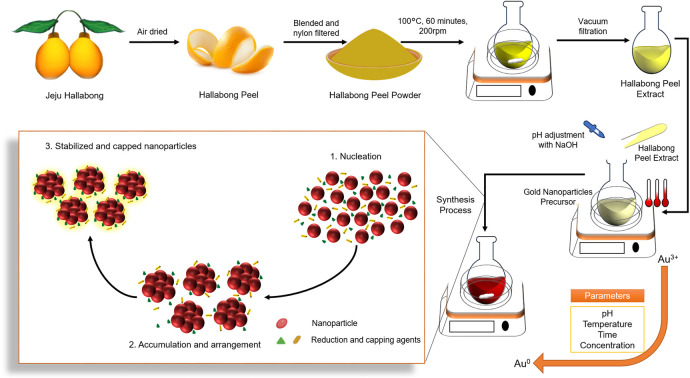

## 1. Introduction

Metallic nanomaterials have gained significant popularity in recent years due to their unique properties in comparison to their bulk form. These materials have shown great potential in various applications, including personalized healthcare, biosensors, diagnostics, and therapies.^1,2^ The scientific community has made rapid progress in developing various types of functional nanoparticles with at least one dimension in the 1–100 nm range.^3^ Among these nanoparticles, gold nanoparticles (AuNPs) have garnered significant attention due to their remarkable optical, electrical, and chemical properties. Additionally, AuNPs have been designed for targeted delivery of anticancer agents.^4–6^ Moreover, functionalizing the surface of AuNPs with a specific purpose, observing the optical characteristics offered by spectrum spectroscopy, and controlling the stability of AuNPs have led to their multiple applications in bioscience and biomedical applications, such as bio-instrumentation, disease diagnosis, drug delivery, gene therapy, and biosensors.^2,7–9^

The development of a synthesis method that is both uncomplicated, adaptable, and environmentally conscious for the production of AuNPs with specific sizes and shapes is a significant and demanding task.^5^ Modifying the synthesis conditions may potentially yield nanoparticles of varying sizes and shapes.^10^ The chemical reduction method utilizing sodium citrate (Na_3_C_6_H_5_O_7_) is the most commonly employed procedure for synthesizing AuNPs, involving the reduction of Au^3+^ to Au^0^ and the stabilization of the resultant nanoparticles.^1,4,5,11^ The process of synthesizing AuNPs through this particular method presents certain limitations, including the presence of unreacted by-products or residual reagents in the solution, which can result in biological toxicity.^12^ As a result, chemically synthesized AuNPs are not ideal for use in biological applications.^7,11^ Additionally, the environmental implications and cost-effectiveness associated with this method have led researchers to explore alternative routes for producing AuNPs. The biosynthesis route, also known as green synthesis, is a popular method among researchers for obtaining AuNPs due to its utilization of environmentally friendly and non-toxic reducing and stabilizing materials, particularly in biomedical applications.^13–15^ However, the production of AuNPs with desired properties and sizes remains a challenge, and the primary functional groups responsible for the reduction and stabilization processes in AuNPs biosynthesis are not yet fully understood.^16^ Consequently, further investigation into this procedure remains an intriguing area of research for researchers.

In general, the green synthesis method, also known as biosynthesis, employs natural resources such as microorganisms, viruses, fungi, algae, and plant extracts to facilitate the production of nanoparticles.^17–21^ Plant extracts, in particular, have demonstrated efficacy as stabilizing and capping agents in the synthesis of AuNPs. This is attributed to the presence of bioactive compositions such as sugars, phenolic compounds, alkaloids, flavonoids, green terpenoids, and proteins, which aid in the synthesis of AuNPs with desirable characteristics such as fine shapes, size, and biocompatibility.^22–25^

The Hallabong, a citrus fruit that is a hybrid of orange and mandarin, is a prominent crop on Jeju Island in South Korea.^26^ This fruit is a relatively recent addition to the citrus family and was named after Halla Mountain. The cultivation of Hallabong fruit is conducted on a significant scale, resulting in the generation of a substantial amount of peel, which is primarily considered a by-product and subsequently disposed of as waste. The peel of Hallabong is known to possess phytochemicals, including flavonoids, alkaloids, and phenolic compounds, which serve as bioactive compositions for the reduction and stabilization of AuNPs in green synthesis.^27^ Furthermore, scientific investigations have demonstrated that orange peels contain health-promoting constituents that exhibit anticancer properties by inhibiting the proliferation of cancerous cells in humans.^28,29^

**Scheme 1:**
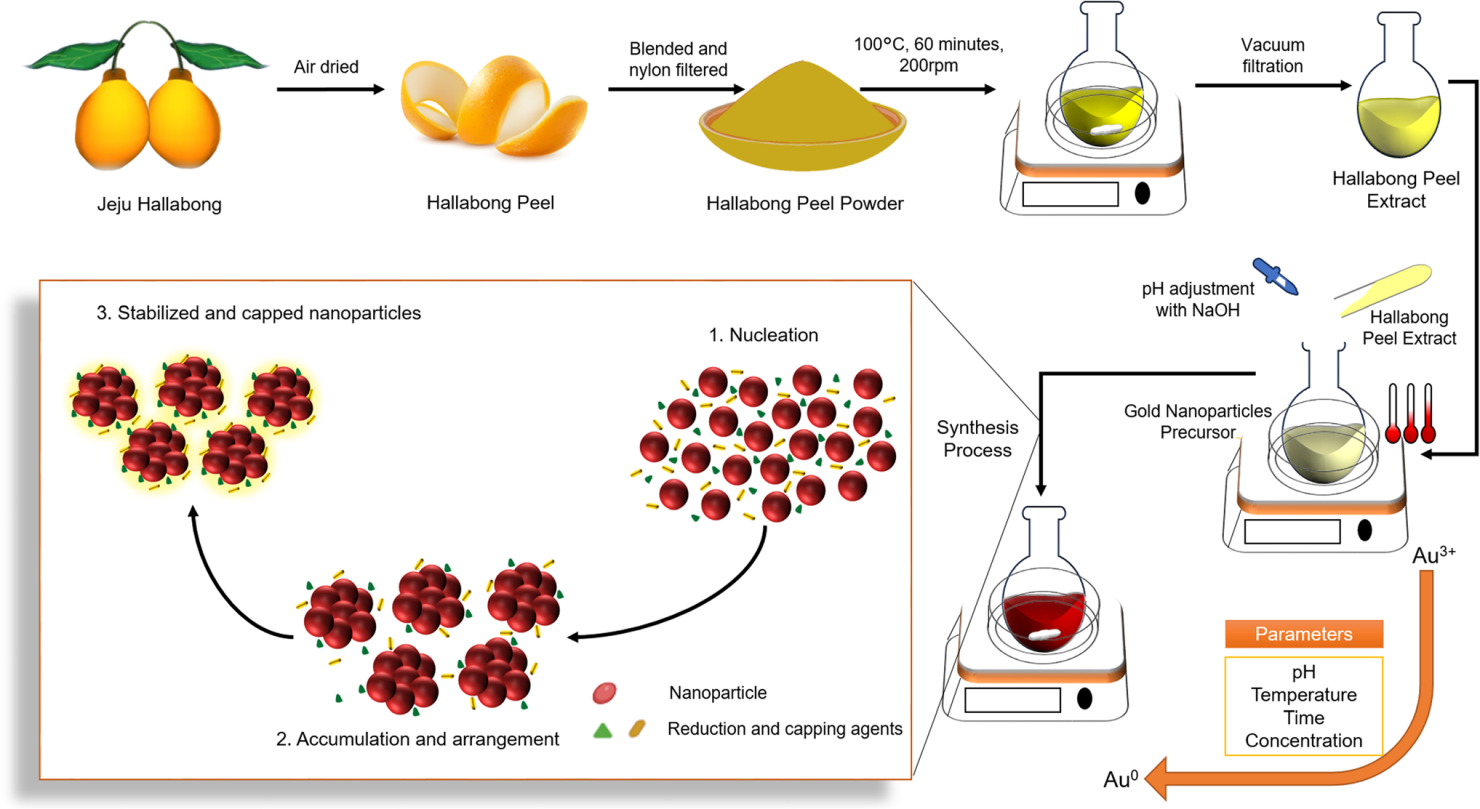
The scheme involves the utilization of Jeju Hallabong peel extract as both a reduction and capping agent in the green synthesis of gold nanoparticles.

While research has been conducted on the chemical makeup and potential health advantages of Hallabong fruit peel extract, there is currently a lack of literature regarding the utilization of said extract to produce gold nanoparticles and their impact on cellular activity. The objective of this study was to explore the capacity of Hallabong fruit peel extract as a reducing and capping agent in the eco-friendly synthesis of AuNPs for the first time. The impact of pH solutions and environmental temperatures on the green synthesis process is being examined in order to determine the optimal outcome. Scheme 1 depicts the complete synthesis protocol. Ultimately, *in vitro* assays are performed to evaluate the optical and biological properties of gold nanoparticles synthesized through green methods as a viable substance for diverse medical uses.

## 2. Results and discussion

**Fig. 1:**
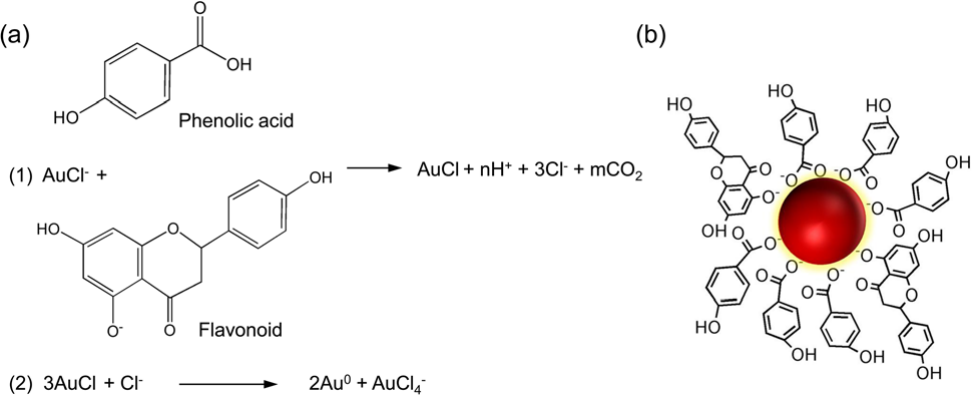
(a) Mechanism underlying the formation and stabilization of gold nanoparticles (b) gold nanoparticle capping by HPE.

The present study reports the successful synthesis of gold nanoparticles (HAuNPs) using gold chloride salt (HAuCl_4_·3H_2_O) and their stabilization with Hallabong peel extract (HPE). The Hallabong peel is known to contain a range of organic compounds, including flavonoids, alkaloids, and phenolic compounds, which have been found to contribute to the synthesis of gold nanoparticles (Fig. 1).^21,30–32^ These compounds are believed to play a crucial role in the synthesis process. The reduction of gold chloride salt (Au^3+^) to gold nanoparticles (Au^0^) is carried out by the donation of electrons or hydrogen atoms in the first step, which requires the presence of a phenolic compound as a reduction agent by releasing electrons from its phenol group in the gold chloride salt solution.^11^ Following that, a process of nucleation and growth of nanoparticles will occur, with the gold atoms created by these reduction processes providing as the nucleation nuclei for the formation of the nanoparticles. The gold nanoparticles are formed when the gold atoms bind together and stack to form bigger crystalline formations.^33,34^ Moreover, flavonoid molecules function as agents for covering surfaces. To stop agglomeration and keep the nanoparticles dispersed in the solution, they form a ligand, or a protective layer around the gold nanoparticles' surface.^4,21^ The solution's color changes during the final stage, which serves as an indicator of the formation of AuNPs. Gold nanoparticles and phenol or flavonoid compounds can interact through covalent or non-covalent bonds that resemble hydrogen or van der Waals-style bonds.

### 2.1 Characterization of Hallabong peels extract (HPE)

**Fig. 2:**
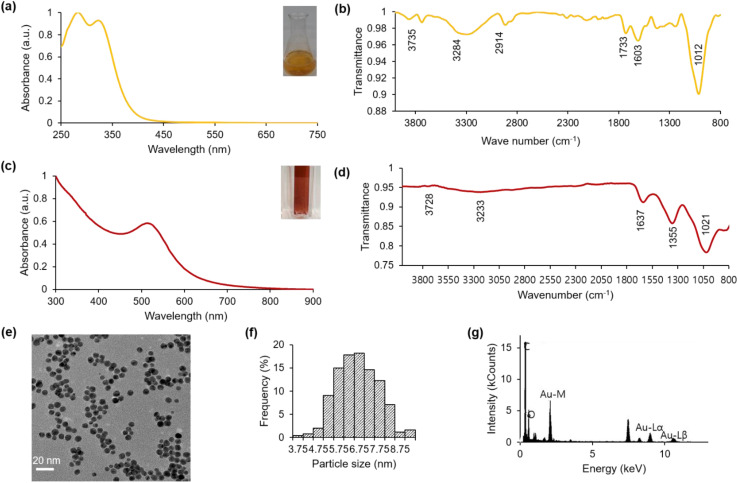
The utilization of Hallabong peel extract was analyzed through (a) UV-visible spectroscopy of HPE; the inset figure included in the text depicts a visual representation of an extract derived from Hallabong peel with a concentration of 10% v/w (b) FTIR spectroscopy. HAuNPs were characterized using various techniques, including (c) UV-visible spectroscopy; the inset figure presented a visual representation of the observed characteristics of HAuNPs. (d) FTIR spectroscopy (e) TEM image (f) particle size distribution from ImageJ software, and (g) EDS image.

The characterization of the Jeju Hallabong peel extract was conducted utilizing diverse analytical techniques. The phytochemicals contained within the extract were identified through qualitative analysis and by referencing reported methods. The UV-visible spectra presented in Fig. 2a reveal that the absorbance peak observed at 284 nm signifies the existence of the phenolic group and other functional groups,^35^ while the absorbance peak at 326 nm is associated with the flavonoid compound present in Hallabong peel extract.^21^ Additionally, the FTIR measurements depicted in Fig. 2b demonstrate that the IR spectrum at wavelengths 3735 cm^−1^, 3284 cm^−1^, 2914 cm^−1^, 1733 cm^−1^, 1603 cm^−1^, and 1012 cm^−1^ correspond to –NH_2_, –OH, –C–H, –C=O, –C=C–, and C–OR functional groups, respectively.^20,32,36–39^ This IR spectrum suggests that the organic compound in Hallabong peel extract contains phenolics, alkaloids, flavonoids, carboxylic acids, *etc.*, and can serve as a reducing and capping agent for the synthesis of gold nanoparticles.^13^

### 2.2 Characterization of biosynthesis of gold nanoparticles (HAuNPs)

Typically, the solution of gold chloride salt (Au^3+^) exhibits a nearly colorless appearance. Upon the addition of Hallabong peel extract (HPE) to the solution under specific pH and temperature conditions, a noticeable alteration in the color of the mixture was observed. The solution transitioned from a light brown color to a dark brown shade, ultimately ending in a red wine coloration at the end of the process. The successful synthesis process of gold nanoparticles capped by Hallabong peel extract (HAuNPs) was indicated by the formation of a red wine colloidal solution.^10,40^ The color formation was regularly monitored and analyzed using UV-visible spectroscopy, which is a convenient technique for validating the formation and stability of AuNPs in an aqueous reaction mixture as shown in Fig. 2c. The findings demonstrated a positive correlation between the peak absorbance observed at 517 nm and the specific range of gold nanoparticles, as reported in other studies.^41,42^

Furthermore, the Fourier transform infrared (FTIR) spectrum displayed alterations in band positions subsequent to the synthesis procedure as shown in Fig. 2d. A spectral shift was detected from the maximum intensity at 1012 cm^−1^ to 1021 cm^−1^. An additional transition was verified to have occurred, whereby the maximum intensity shifted from 3284 cm^−1^ to 3233 cm^−1^, and from 3735 cm^−1^ to 3728 cm^−1^. The merging of two peaks observed at bands 1733 cm^−1^ and 1603 cm^−1^ from HPE resulted in a single peak at band 1637 cm^−1^ in HAuNPs. Subsequently, a minor abrupt elevation in the range of 1200–1400 cm^−1^ observed in the Hallabong peel extract (HPE) transformed into a distinct and well-defined peak at 1355 cm^−1^ in the case of HAuNPs. Thus, it is suggested that the modified peaks observed in the FTIR analysis of the nanoparticles can be attributed to the formation of nanoparticles synthesized by HPE. The phenolic compounds and flavonoids present in HPE have been observed to actively reduce metal ions through the oxidation of aldehyde to carboxylic acid.^3,43,44^ The stabilizing function of these particular functional groups is widely recognized in academic literature.^45,46^ The emergence of a novel medium peak with sharp characteristics at 1355 cm^−1^ could potentially be attributed to the stretching vibrations of C–H, while the previously observed blunt peaks have dissipated. An observation was made that an increase in wave numbers led to a significant increase in the absorption bands of AuNPs. The coordination bonds established between AuNPs and functional groups (O–H, C=O) may facilitate this outcome.^47^ The FT-IR spectrum serves to demonstrate HPE's potential as a reducing, stabilizing, and capping agent.

Additionally, a TEM analysis was performed to investigate the morphology and size of the nanoparticle following the synthesis procedure. Fig. 2e shows the TEM image of HAuNPs. The transmission electron microscopy analysis reveals a clear appearance of gold nanoparticles with spherical morphology. The visual representation depicts particles exhibiting a high degree of uniformity and good dispersion. The utilization of ImageJ software for analysis revealed that the mean size of HAuNPs was approximately 7 nm, as represented in Fig. 2f. Subsequently, Fig. 2g shows the EDS image obtained from HAuNPs. Upon conducting result analysis, it was observed that there existed a robust signal at the 2.120 keV peak, which corresponded to the presence of elemental gold in the biosynthesized nanoparticles. The existence of elemental gold effectively facilitated the appearance of the peak in the absorption spectrum of UV-visible spectra. In addition to the robust signal resulting from the elemental gold, a potent signal is also detected from the C element, which we suggested originates from the carbon grid utilized for analytical purposes. Subsequently, a number of weak signals were detected, specifically oxygen and nitrogen, emanating from X-ray discharges originating from biomolecules affixed to the exterior of Au nanoparticles. The NaOH solution also serves as a source of Na atoms.^16^

### 2.3 The impact of pH on the green synthesis of HAuNPs utilizing HPE

**Fig. 3:**
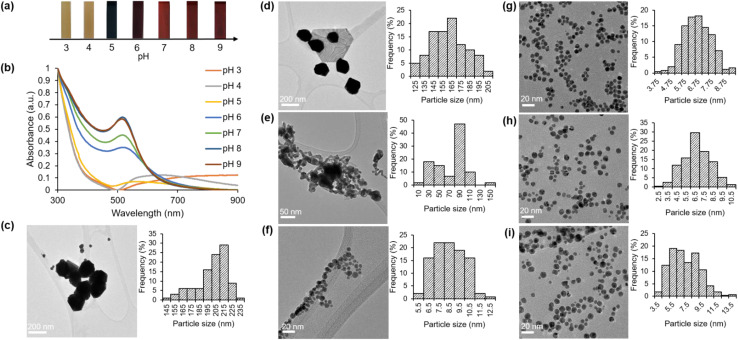
(a) The visual observation of gold nanoparticles (HAuNPs) under varying pH conditions (b) UV-visible spectroscopy results of HAuNPs at different pH levels. Transmission electron microscopy (TEM) images of gold nanoparticles (HAuNPs) using HPE and corresponding particle size histograms are presented for various pH values at (c) pH = 3 (d) pH = 4 (e) pH = 5 (f) pH = 6 (g) pH = 7 (h) pH = 8 (i) pH = 9.

The objective of this investigation was to examine the impact of pH on the formation of gold nanoparticles through the utilization of a green synthesis approach. The mixture solution of gold chloride salt and HPE was subjected to various pH conditions (ranging from 3 to 10) prior to the start of the reaction through the addition of varying quantities of OH^−^. Our study focused on the examination of various appearance characteristics of the green synthesis gold nanoparticles (HAuNPs), including their shape, size, surface charge, and distribution. The visual manifestation of varying pH levels in the synthesis process is portrayed in Fig. 3a. Under acidic conditions, the resultant solution exhibited a small alteration in color when compared to the initial color of the mixture. Following the increase in pH, a notable alteration in color was detected. Under pH conditions ranging from pH 6 to 10, the tone of the color is perceptually indistinguishable. Moreover, in order to examine this phenomenon, UV-visible spectroscopy was conducted for analysis. Fig. 3b exhibits the absorbance curves of the obtained HAuNPs.

The absorbance peak within the specified range for gold nanoparticles is not observable at pH 3 and pH 4. The present findings have led us to infer that the process of synthesis did not take place under the given pH condition. The UV-visible spectrum obtained at a pH of 5 exhibited a significantly wide absorbance with a maximum wavelength of 570 nm. The outcome of this experiment yields a linear structure that exhibits a visually perceptible dark purple color within the solution. A noticeable alteration was detected at a pH of 6, wherein the absorbance peak within the designated range for gold nanoparticles was evident (*λ*_max_ = 518 nm). A blueshift was observed in the absorbance peak, shifting from 518 nm to 513 nm, as the pH of the solution increased from 6 to 9. The phenomenon observed is that the size of HAuNPs decreases as the pH of the solution increases. pH changes can have an impact on the physical and chemical structure of gold nanoparticles, which can then change their surface charge and the effective dielectric environment surrounding them.^48,49^ These changes can also have an impact on the optical characteristics of the gold nanoparticles, their surface plasmon resonance frequency, their surface load, and their electrostatic interactions with the surrounding compounds.^50^ Each of these factors also affects how a particle interacts with its surroundings, which in turn effects how the particle size distribution appears on the UV-vis absorption spectrum. Table 1 presents a positive correlation between the absorbance intensity and the pH level of the solution.

**Table 1:** Summary of the characterization outcomes of HAuNPs in response to changes in pH conditions

pH	Absorbance peak (nm)	Hydrodynamic diameter (*d*, nm)	Zeta potential (mV)	Average particle size (nm)
3	NA	191.30	−21.7	200.76
4	NA	188.40	−24.6	161.74
5	570	72.10	−26.3	73.75
6	518	19.35	−34.9	8.48
7	517	18.52	−32.1	6.67
8	516	16.50	−30.5	6.70
9	514	12.87	−32.0	7.15

The utilization of dynamic light scattering (DLS) was employed to examine the size distribution and intensity of nanoparticles across varying pH conditions. The graphs (Fig. S1a–g†) illustrate those particles with diameters ranging from 80 to 400 nm were observed at lower pH levels (3 and 4). Specifically, particles with diameters of approximately 191.3 nm and 188.4 nm were observed at pH levels 3 and 4, respectively. The reduction in particle diameter was observed with an increase in pH conditions, which was supported by the UV-visible spectroscopy findings. At a pH value of 5, the size range of particles is within the range of 20 to 200 nm. Subsequently, within the pH range of 6 to 9, the size of the particles ranges from 6 to 20 nm in diameter. Consequently, the TEM images reveal a slightly higher presence of polydisperse particles. Table 1 presents a comprehensive overview of the diameter values of concern.

In addition, we assessed the electric charge present on nanoparticles, commonly referred to as the zeta potential (*z*), which provides critical information regarding the stability of nanoparticle dispersion as determined by dynamic light scattering (DLS). The zeta potential serves as an indicator of the existence of repulsive forces and can be utilized to assess the stability of nanoparticle dispersions over an extended duration.^44,51^ The stability of the scattering of nanoparticles is determined by the equilibrium between attractive and repulsive forces that exist among them.^52,53^ A stable particle dispersion is characterized by the presence of similar repulsion among the particles.^54^ Conversely, if there is an absence or minimal repulsion among the particles, aggregation will occur. The HAuNPs exhibit zeta potential values ranging from −21.7 to −34.9 mV, as shown in Fig. S2a–g.† A zeta potential value that is negative results in the repulsion of particles, indicating a lack of attraction towards aggregates.^55^ This is in accordance with others' findings.^22–25^

Further, TEM studies were conducted to investigate the morphology and particle size under varying pH conditions. The TEM images presented in Fig. 3g–i demonstrate that the HAuNPs exhibit a homogeneous distribution and spherical morphology when the pH is elevated within the range of 7 to 9. The experimental results showed no aggregation of HAuNPs, and the particle size remained at the nanoscale level. Large particle sizes were observed at pH values of 3 and 4 (Fig. 3c and d). At a pH of 5 (Fig. 3e), a reduction in particle size is observed; however, there is also evidence of aggregation. The process of aggregation exhibited a decline upon reaching a pH of 6 (Fig. 3f), while the dimensions of the particles remained consistently reduced. The findings of the study indicate that HAuNPs' size and size distribution can be regulated by utilizing hydroxyl ions and varying the addition of NaOH.^3,16^ The ideal quantity and concentration of hydroxyl ions can restrict the growth of small nanoparticles and enhance their size distribution. Furthermore, we present the histogram generated by the ImageJ software to analyze the mean particle size of the HAuNPs next to the TEM image for each pH condition. As illustrated in Fig. 3 and presented in Table 1, it can be observed that an increase in pH results in the stabilization of the average particle size at approximately 6.6 to 7.1 nm in diameter.

### 2.4 The impact of temperature on the green synthesis of HAuNPs using HPE

**Fig. 4:**
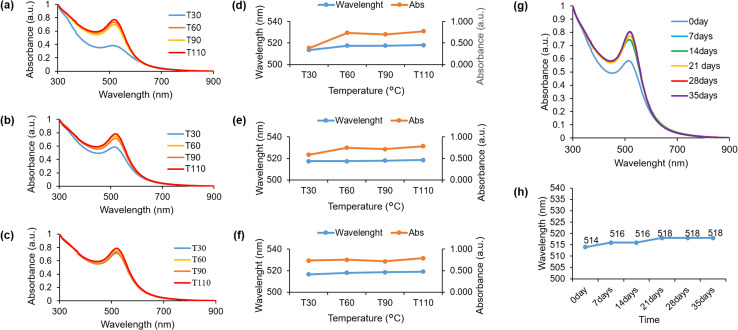
The UV-visible spectroscopy outcomes of the HAuNPs in a reaction with varying temperatures are presented as follows: (a) 30 minutes (b) 120 minutes reaction time and (c) 24 hours after synthesis process. Changes in the absorption peak and the intensity of the absorption peak on (d) 30 minutes (e) 120 minutes reaction time and (f) 24 hours after synthesis process. (g) The stability of colloidal gold nanoparticles (AuNPs) capped with HPE was demonstrated through the UV-visible spectroscopy analysis of the obtained results. (h) Peak absorbance value of the HAuNPs in each measurement.

The impact of temperature on the green synthesis process utilizing HPE was investigated by manipulating the temperature conditions throughout the reaction. UV-visible spectroscopic analyses were conducted at 30 minutes intervals until the completion of the stirring process, which occurred within a 2 hours timeframe. The present study employed a pH of 9 for the synthesis of HAuNPs, with the aim of examining the impact of reaction temperature at four different conditions, which is 30 °C, 60 °C, 90 °C, and 110 °C. During the initial half-hour of the synthesis process at a reaction temperature of 30 °C, the HAuNPs colloidal exhibited smaller absorbance peaks (513.5 nm) compared to other samples. However, the intensity at the absorbance peaks was significantly low, as shown in Fig. 4a. Upon concluding the reaction after a duration of 2 hours, it was observed that the colloidal absorbance peak of HAuNPs at 30 °C underwent a redshift (517.5 nm), resulting in a negligible difference in its value when compared to the absorbance peak at an elevated temperature as shown in Fig. 4d. Although the intensity of the absorbance peak demonstrates an increase, it remains significantly lower in comparison to the absorbance peak intensity value observed at an elevated reaction temperature, as depicted in Fig. 4b. In addition, the HAuNPs colloidal samples were subjected to UV-visible spectroscopy analysis subsequent to 12 hours cooling period at room temperature. The absorbance peaks of HAuNPs colloidal samples exhibit stability within the 516.5–519 nm range, as evidenced by Fig. 4c. However, the absorption peak intensity noticeably increases at a reaction temperature of 30 °C, resulting in an equivalent value to that of the other samples. The findings suggest that a reduction in temperature during the synthesis process facilitates a gradual nanoparticle preparation process, leading to a reduced peak absorbance compared to a rapid reaction at an elevated temperature (Fig. 4d–f). As such, it can be inferred that the temperature of the synthesis process has an impact on the characteristics of the resulting nanoparticles. Consequently, the augmentation in the intensity of the absorbance peak is indicative of a rise in the concentration of HAuNPs in the colloidal, as illustrated in Fig. S3a.† The absorbance peak intensity in Fig. S3b–d† remains constant during the initial 30 minutes of the reaction and for the subsequent 12 hours after the completion of the process. While the absorbance peak of HAuNPs at a higher reaction temperature exhibits a slightly greater magnitude compared to that of HAuNPs at a lower reaction temperature, the duration of the reaction is significantly reduced. The utilization of this method has the potential to reduce the duration of the synthesis process. The hydrodynamic diameter and zeta potential of all colloidal samples of HAuNPs were measured after 12 hours cooling period at ambient temperature. The findings indicate a positive correlation between rising temperature and an increase of hydrodynamic diameter. However, it is noteworthy that the outcome exhibits a decline at a temperature of 110 °C, as shown in Fig. S4.† Analogous trends are also observed in the determination of potential zeta potentials; however, all values continue to exhibit favourable particle dispersion. The manipulation of reaction temperature enables regulation of the absorbance peak of the AuNPs, which is indicative of the size of the particles, and the duration of the synthesis process.

### 2.5 The stability of HAuNPs

The investigation evaluated the stability of gold nanoparticles that were synthesized and capped with HPE. This was accomplished by analyzing the absorbance peak using UV-visible spectroscopy analysis for a duration of 35 days. Weekly measurements were taken. Fig. 4g and h illustrates a marginal redshift ranging from 514 nm to 518 nm (day 0 to day 35). Subsequent to this, the peak of absorbance exhibits a consistent state for a duration of 35 days. Moreover, there was no notable alteration of particle size, and there is no visible alteration in color or aggregation observed, which suggests the stability of the synthesized gold nanoparticles.

### 2.6 Colorimetric test

It is well known that gold nanoparticles have a strong affinity for biomolecules including proteins, peptides, antibodies, oligonucleotides, and pathogens.^56,57^ Thus, functionalizing gold nanoparticles with these biomolecules enables their use as biomarkers in detection applications.^57,58^ It is of great interest to develop biomarker/biosensor applications based on nanoparticle aggregation due to the unique physicochemical properties of gold nanoparticles, such as their ability to change color due to plasmon coupling between particles. In this study, we investigated the potential of our gold nanoparticles synthesized using Hallabong fruit peel extract for biosensor applications through a colorimetric assay. The research from Pambudi *et al.*^59^ and Lismont *et al.*^60^ utilizes the interaction between biocytin and avidin to prepare a colorimetric assay based on Au–citrate and they found that the biocytin–avidin complex induces a cross-linking aggregation and leads to optical properties modification of gold nanoparticle. Avidin is composed of four identical subunits and is capable of forming a highly stable non-covalent complex with biocytin.^61,62^ The biocytin–avidin complex typically possesses a massive interaction and the extraordinary affinity of biocytin for avidin (*K*_a_ = 10^−15^ M^−1^),^63^ so avidin–biocytin non-covalent bonding is specific, extremely strong, and can be extremely useful in biosensor applications. Therefore, we used the biocytin–avidin interaction in this colorimetric assay to evaluate our gold nanoparticles.

The functionalization of HAuNPs is a crucial phase in the preparation of the probe for the colorimetric sensor. Coupling mechanism from the presence of amine-containing lysine moieties in biocytin towards AuNPs was used. EDC, a water-soluble carbamate, is used to modify the surface of HAuNPs to make the carboxyl group reactive with primary amines. The active ester compound NHS (*N*-hydroxy-succinimide) can be employed to create amide bonds when the primary amine is present on the surface of the HAuNPs.^64^ In this study, we were successful in forming stable biotinylated-HAuNPs. There was no difference in color and peak absorbance in any sample significantly, when compared to HAuNPs alone (Fig. S5†).

**Fig. 5:**
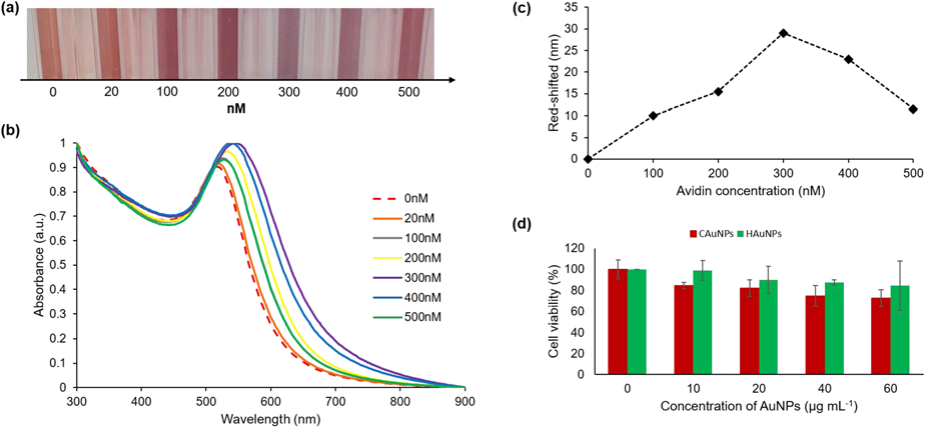
(a) Visual observation (b) UV-visible spectroscopy analysis (c) shifting value in colorimetric assay of biotinylated-HAuNPs with various concentrations of avidin, (d) the impact of varying concentrations of CAuNPs and HAuNPs on the cell viability of the RAW 264.7 cell line.

The colorimetric assay of HAuNPs disclosed here shows the sensor's capacity to detect avidin with observable color variations down to the nanomolar range. Changes in colors and shifts in the absorption peaks can be used to identify changes in the optical characteristics of the biotinylated-HAuNPs solution. Lower avidin concentrations did not significantly alter the color, as seen in Fig. 5a. A considerable change from red to light purple in biotinylated-AuNPs solution color was seen when avidin concentration was increased. After that, the color of the biotinylated-AuNPs solution did not significantly alter at a particular avidin concentration point. This visual observation is consistent with the analysis's UV-visible spectroscopy results, which are displayed in Fig. 5b. Following an increase in avidin concentration, the absorbance peak redshifted and broadened. We refer to the avidin concentration as the optimum avidin concentration when it results in the highest redshift peak absorbance of the biotinylated-HAuNPs solution when compared to the biotinylated-HAuNPs solution without avidin as a control. The peak absorption shifts from 516 nm to 545 nm at a concentration of avidin around 300 nM, as seen in Fig. 5c. At particular points with a higher concentration, the peak absorption then experiences a blue shift.

**Scheme 2:**
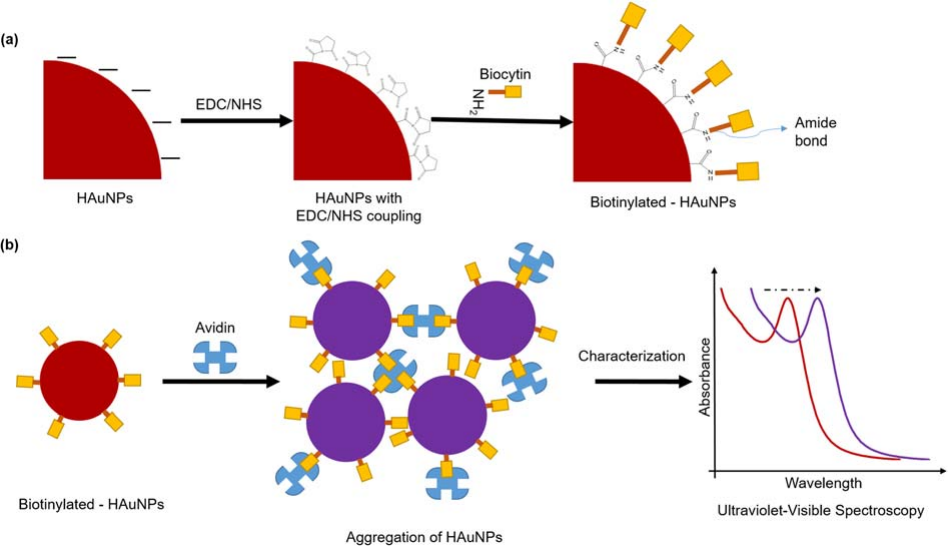
(a) Surface modification by EDC/NHS and functionalization procedure by biocytin of HAuNPs. (b) The colorimetric assay procedure of biotinylated-AuNPs using avidin.

As previously explained, avidin is made up of four identical tetrametric proteins that can form a maximum number of cross-links with a total of four biocytins. As seen in Scheme 2b, this cross-linking can lead to aggregation. The biotinylated-HAuNPs solution's color and absorbance spectrum are only a little altered by aggregation since it doesn't occur extensively at low concentrations. A huge amount of aggregation happens when avidin concentration rises, increasing the cross-linking between avidin and biocytin. This significantly impacts the color and absorbance spectrum of the biotinylated-HAuNPs solution. A further rise in avidin concentration, however, had no discernible impact on the color and absorbance spectrum of biotinylated-HAuNPs in comparison to the control. Steric hindrance can explain this behavior. Large amounts of avidin will completely cover all of the active sites of biocytin when added to a solution of biotinylated-HAuNPs, threatening aggregation. Accordingly, an ideal level of avidin concentration can produce the optimum possible interaction between avidin and biocytin, which results in an optimal level of both a steric hindrance and the aggregation of nanoparticles. The experimental findings in this work also coincided with those of studies by Pambudi *et al.*^59^ and Lismont *et al.*,^60^ which likewise employed AuNPs produced through chemical reduction. For that reason, this study presents preliminary analyses of potential sensor tunability employing AuNPs produced through a green synthesis method.

### 2.7 Cell viability test

The cytotoxicity and potential of HAuNPs for biomedical applications were evaluated through the implementation of an MTT assay. This study investigates the biocompatibility of AuNPs with the widely used murine macrophage cell line RAW 264.7. The results obtained from Fig. 5d indicates that HAuNPs exhibit favorable biocompatibility characteristics across all concentrations in comparison to CAuNPs. The impact of HAuNPs at concentrations of 10 μg mL^−1^, 20 μg mL^−1^, 40 μg mL^−1^, and 60 μg mL^−1^ on cell viability was assessed, revealing corresponding values of 98.94%, 90.05%, 87.59%, and 84.49%. The concentration-dependent increase in toxicity is observed in cells exposed to higher concentrations of HAuNPs. The cellular viability was found to be the lowest in the presence of CAuNPs. As the concentration of CAuNPs increased, the observed cell viability decreased to 84.49%, 82.05%, 74.70%, and 72.35%, respectively. The findings indicate that the utilization of natural reducing and capping agents from Hallabong peel extract for the synthesis of gold nanoparticles yields superior biocompatibility properties and low cytotoxicity properties in comparison to chemically synthesized gold nanoparticles. This approach exhibits promising prospects for the development of biomedical applications.

## 3. Experimental

### 3.1 Materials

The Hallabong fruits were procured from a marketplace located in Daejeon, South Korea. Hydrogen tetracholoroaureate(iii) hydrates (HAuCl_4_·3H_2_O, 99.9%), avidin, biocytin, *N*-hydroxysuccinimide (C_4_H_5_NO_3_ MW = 115.09 g mol^−1^), and NaOH were purchased from Sigma Aldrich (St. Louis, MO, USA). 1-(3-Dimethylaminopropyl)-3-ethylcarbodiimide hydrochloride (C_8_H_17_N_3_–HCl MW = 191.70 g mol^−1^) purchased from Tokyo Chemical Industry Co., Ltd (Toshima, Kita-Ku, Tokyo, Japan). RAW 264.7 cell lines were purchased from ATCC (American Type Culture Collection). The experiment utilized distilled water as the primary solvent.

### 3.2 Preparation of Hallabong fruit peel extract (HPE)

Hallabong fruit peels were washed thoroughly with running water to remove dirt and then dried at room temperature for 3 days. Then the peels were cut into pieces using a blender and filtered through a nylon filter to obtain peel powder. After that, the HPE was made by mixing and stirring 10% w/v of Hallabong fruit peel powder and DI water while heated to boiling temperature for 60 minutes. After cooling the solution to room temperature, it was subjected to filtration using Whatman filter paper no. 2 and subsequently centrifuged at 12 000 rpm for a duration of 20 minutes. The HPE solution was obtained by filtering the supernatant through a 20 μm syringe filter. Subsequently, the HPE solution was preserved at a temperature of 4 °C to facilitate its subsequent utilization.

### 3.3 Biosynthesis of gold nanoparticles (HAuNPs)

In a round-bottom flask, 1 mM of chloroauric acid solution (HAuCl_4_·H_2_O) and 10 : 1 of HPE solution were mixed, respectively, by volume (% v/v). Then, the pH of the mixed solution was adjusted before stirring at 200 rpm at a certain temperature for 2.5 hours to aid the complete reduction of gold chloride to AuNPs. Then, a specific quantity of sodium hydroxide (NaOH) is introduced into the mixed solution to regulate the pH level from a value of 3 to 10. Following the completion of the synthesis process, each solution undergoes characterization in order to examine the impact of pH on the previously mentioned procedure.

In addition, the present study involves an investigation of the impact of temperature on the reaction during the synthesis process, using a specific pH value obtained from a previous experiment.

### 3.4 Characterization of the Hallabong fruit peel extract (HPE)

The investigation of organic compounds was conducted using a UV-visible spectrophotometer (Optizen Pop, Mecasys, Daejeon, South Korea) by scanning the extract solution within the wavelength range of 250–700 nm in the UV-visible region. The researchers employed a Fourier transform infrared (FT-IR) spectrophotometer to analyze the extract's functional groups. The extract powder was scanned within the wavelength range of 900–4000 cm^−1^.

### 3.5 Characterization of the biosynthesis of gold nanoparticles

The biosynthesis of AuNPs was characterized through the utilization of multiple instruments. The process of complete reduction of Au^3+^ ions to Au^0^ ions was initially examined using a UV-visible spectrophotometer in the UV-visible range, specifically at a wavelength of 400–800 nm. Deionized water was utilized for the purpose of conducting background correction on UV-visible spectra. The functional groups present in the extract were identified using a FT-IR spectrophotometer, which involved scanning the extract powder within the wavelength range of 900–4000 cm^−1^. The examination of particle size, morphology, and particle distribution was conducted through the utilization of TEM (Thermo Fisher, TALOS F-200X) at 200 kV, located at the Korea Research Institute of Chemical Technology (KRICT) in Daejeon, South Korea. A carbon grid was utilized to deposit a small quantity of HAuNPs, which were subsequently subjected to a drying process. Subsequently, the specimen was relocated for the purpose of capturing visual representations. The acquired images underwent analysis through the use of ImageJ software. The confirmation of the presence of elemental gold was achieved through the utilization of an EDS detector, which was equipped with a TEM instrument. The hydrodynamic diameters of nanoparticles and the surface charge of AuNPs were investigated using dynamic light scattering and zeta potential analysis (DLS; Zetasizer Nano ZS, Malvern Instruments, Malvern, UK) at the National NanoFab Center in Daejeon, South Korea.

### 3.6 Colorimetric assay

The procedure utilized in this colorimetric test follows, with adjustments, on the procedure in a study performed by Pambudi *et al.*^59^ Firstly, EDC/NHS is used to perform surface modification on HAuNPs. EDC/NHS reaction will facilitate a reaction between the amine group in biocytin and the carboxylic group on the surfaces of HAuNPs.^30^ During this procedure, 1 mg mL^−1^ of EDC solution was added to HAuNPs solution, followed by 1 mg mL^−1^ of NHS solution. The mixture was further vortexed to yield a homogenous solution, which was left to incubate for 30 minutes. Then, the 10^−4^ M biocytin solution was added to the HAuNPs solution modified with EDC/NHS and incubated for one hour. Scheme 2a depicts an illustration of the functionalization procedure. Lastly, we evaluate the colorimetric assay performance with the addition avidin as analyte at concentrations ranging from 20 to 500 nM to a solution of biotinylated-HAuNPs and incubating it for at least 30 minutes. Using a UV-visible spectrometer, the samples were characterized. Scheme 2b shows an illustration of the colorimetric assay.

### 3.7 Cell viability test

The main text of the article should appear here with headings as appropriate. The MTT assay is employed to assess the cytotoxicity of nanomaterials by conducting a cell viability test. In order to achieve the objective, RAW 264.7 cell lines were selected and subjected to a 24 hours incubation with AuNPs derived from green synthesis (HAuNPs) and chemically reduced synthesis (CAuNPs). The medium devoid of nanomaterials was employed as a control experiment. The experimental procedure was conducted in triplicate. The optical absorption of a 96-well plate was measured at a wavelength of 450 nm using an ELISA reader. The equation was utilized to ascertain the percentage of cell viability.^65^1
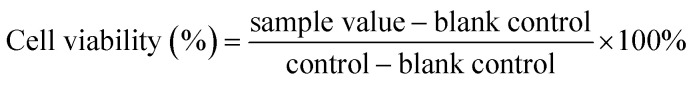


## 4. Conclusions

The present investigation employed a green synthesis approach utilizing Jeju Hallabong peel extract (HPE) for the production of gold nanoparticles. The characterization of HPE was conducted to identify various organic compounds that function as reducing agents in the process of reducing Au^3+^ to Au^0^, thereby facilitating the production of HAuNPs. Additionally, these compounds also serve as stabilizing and capping agents. After a monitoring period of 35 days, the gold nanoparticles exhibited favorable stability. The study examined the impact of varying pH conditions through the introduction and manipulation of OH^−^ concentration in the initial solution. The study revealed that the morphology and particle size of HAuNPs were subject to towards pH levels. Specifically, an increase in pH was observed to facilitate a reduction in nanoparticle sizes, resulting in the formation of uniform spherical nanoparticles characterized by a narrow size distribution. Furthermore, it is possible to optimize the temporal kinetics by modulating the thermal conditions during the synthesis procedure. Elevating the process temperature can accelerate the synthesis duration even though potentially yield nanoparticles of marginally greater dimensions. Moreover, the potential biocompatibility of gold nanoparticles (HAuNPs) presents a favorable outlook for their utilization in the field of biomedical research. The outcomes of an avidin–biocytin colorimetric assay provide preliminary analyses of possible sensor tunability using HAuNPs produced through a green synthesis method. In addition, *in vitro* cytotoxicity assays indicated that the HAuNPs exhibited minimal toxic effects on the RAW 264.7 murine macrophage cell line at various concentrations, in contrast to gold nanoparticles produced *via* chemical synthesis methods.

## Author contributions

Ariski Ridhola Tri: conceptualization, methodology, data curation, visualization, writing-original draft; Kyung Kwan Lee: methodology, data curation, investigation; Yongkwan Kim: format analysis, validation; Chang-Soo Lee: conceptualization, methodology, data curation, investigation, writing-review & editing, project administration, supervision.

## Conflicts of interest

There are no conflicts to declare.

## Supplementary Material

RA-014-D4RA00614C-s001
