# Supplementary material for: The impact of pH and temperature on the green gold nanoparticles preparation using Jeju Hallabong peel extract for biomedical applications
Source: RSC Adv. 2024 May 3;14(21):14582–92. doi: 10.1039/d4ra00614c (PMC11066618; doi:10.1039/d4ra00614c)
Supplement: RA-014-D4RA00614C-s001 [file RA-014-D4RA00614C-s001.pdf]

Electronic Supplementary Material (ESI) for RSC Advances.
This journal is © The Royal Society of Chemistry 2024

Supplementary Information

Effect of pH and Ambient Temperature on Synthesis of Green Gold Nanoparticles Using Jeju Hallabong Peel Extract as a Friendly Approach for Biomedical Applications

Ariski Ridhola Tri,‡[ab] Kyung Kwan Lee,‡[a] Yongkwan Kim[c] and Chang-Soo Lee*[ab]

[a]Bionanotechnology Research Center, Korea Research Institute of Bioscience and Biotechnology (KRIBB), Daejeon, 34141, Republic of Korea; ariskiridh@gmail.com (R.T.A); lkk@kribb.re.kr (K.K.L).

[b]Department of Biotechnology, University of Science & Technology (UST), Daejeon 34113, Republic of Korea.

[c]Wildlife Disease Response Team, National Institute of Wildlife Disease Control and Prevention (NIWDC), Gwangju 62407, Republic of Korea; kyk5388@korea.kr (Y.K).

‡ These authors contributed equally to this paper.

* Correspondence: cslee@kribb.re.kr.

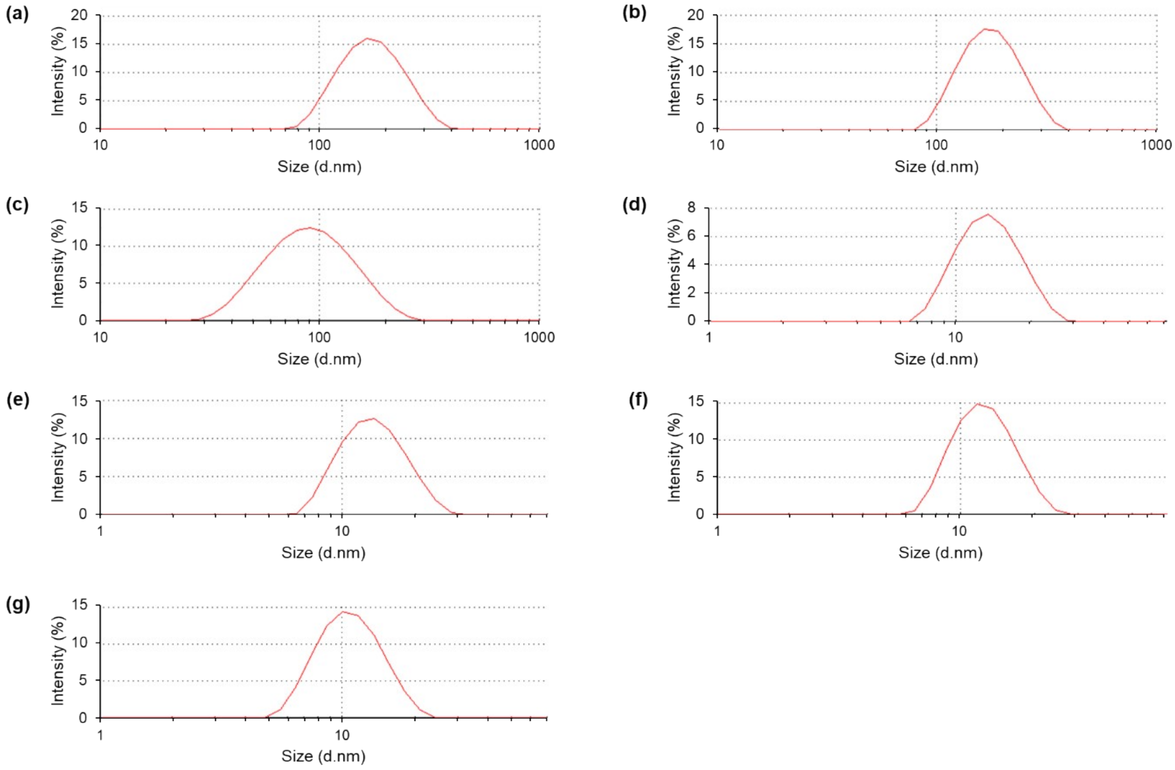

Figure S1. Size distribution images of gold nanoparticles (HAuNPs) that were synthesized using HPE. The images depict the size distribution of AuNPs at varying pH levels: (a) pH = 3; (b) pH = 4; (c) pH = 5; (d) pH = 6; (e) pH = 7; (f) pH = 8; (g) pH = 9.

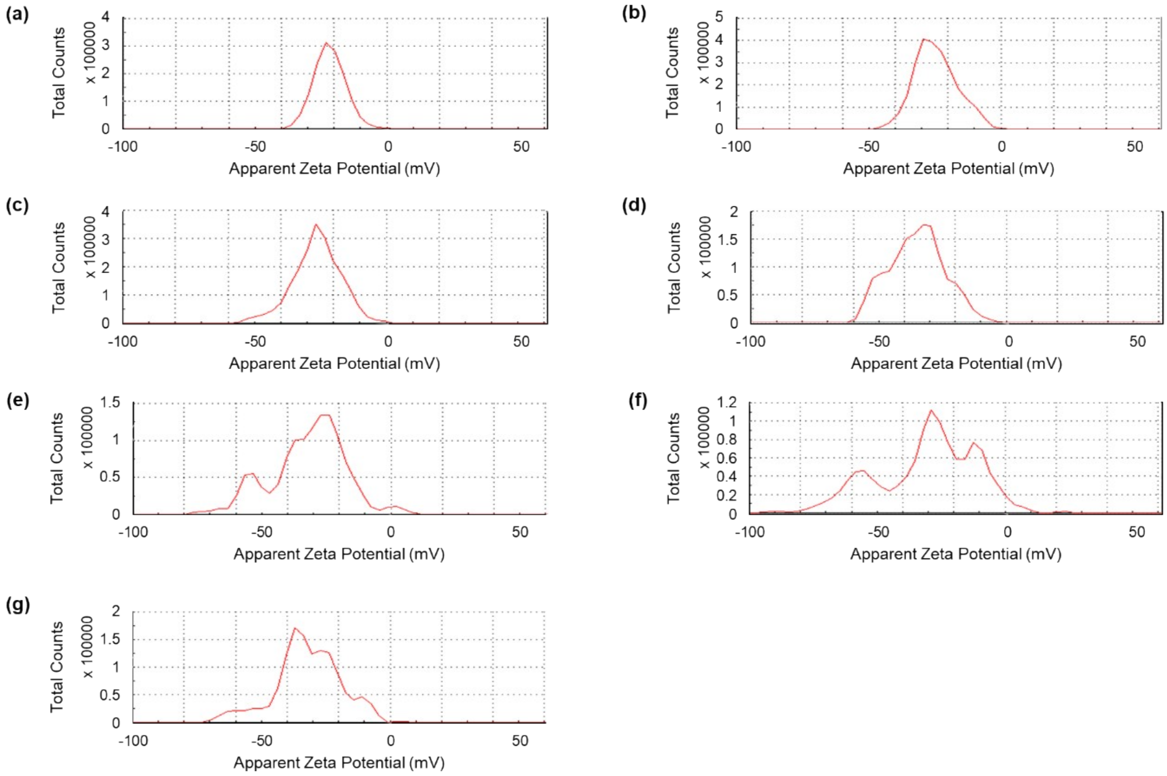

Figure S2. The zeta potential values of gold nanoparticles (HAuNPs) synthesized using HPE were measured at various pH levels: (a) pH = 3; (b) pH = 4; (c) pH = 5; (d) pH = 6; (e) pH = 7; (f) pH = 8; (g) pH = 9.

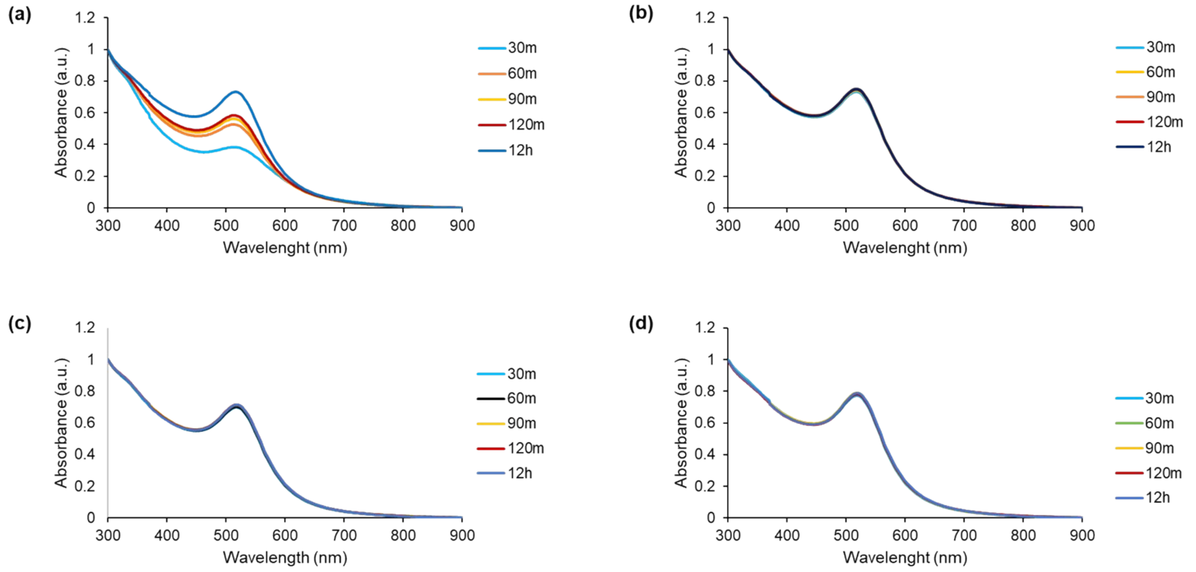

Figure S3. The absorbance peak of HAuNPs at pH 9 and temperature reaction at (a) T = 30 °C, (b) T = 60 °C, (c) T = 90 °C, and (d) T = 110 °C.

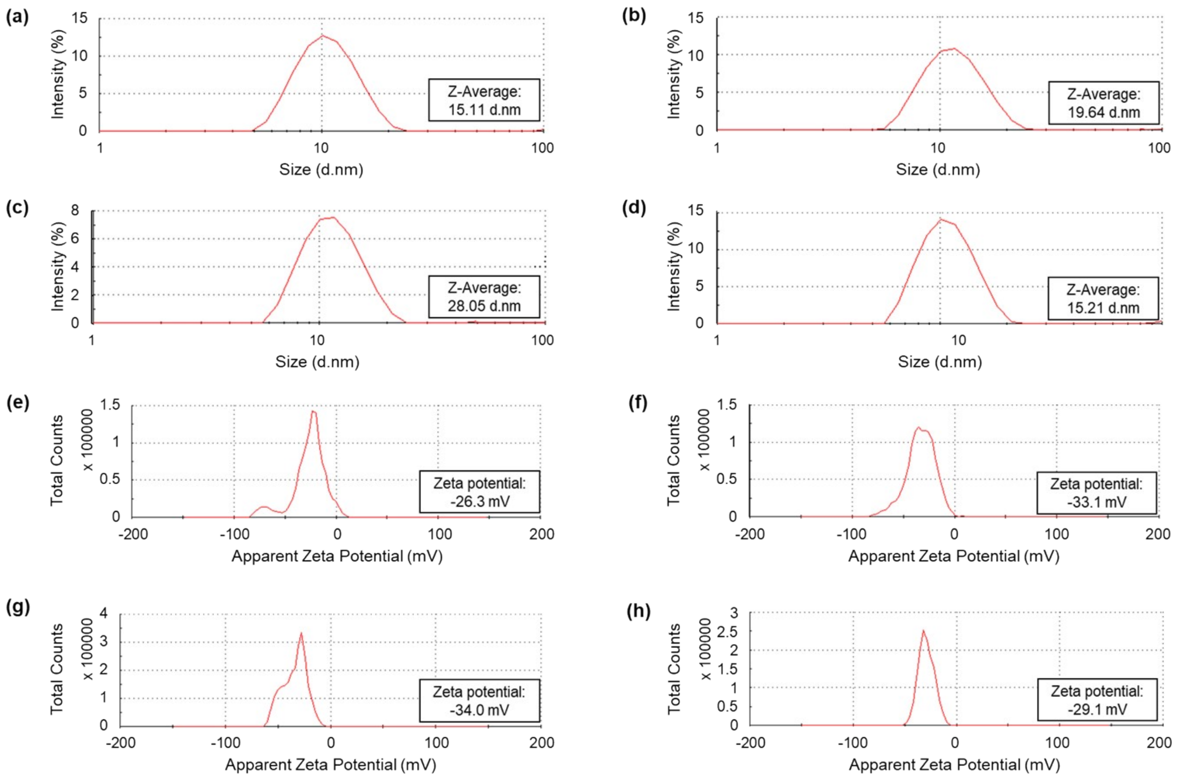

Figure S4. Hydrodynamic diameter of HAuNPs at pH 9 and different temperature reactions: (a) T = 30 °C (b) T = 60 °C (c) T = 90 °C and (d) T= 110 °C. Zeta potential of HAuNPs at pH 9 and different temperature reactions: (e) T = 30 °C (f) T = 60 °C (g) T = 90 °C and (h) T = 110 °C.

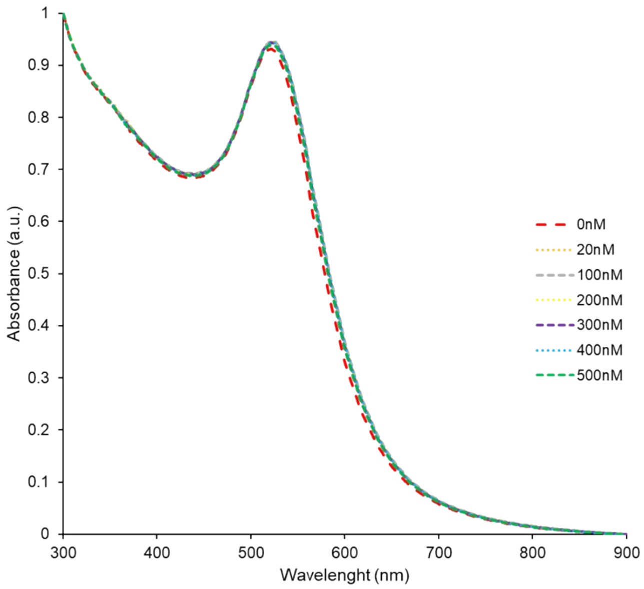

Figure S5. Biotinylated-HAuNPs before being treated with Avidin.
